# Catheter Management and Risk Stratification of Patients With in Inpatient Treatment Due to Acute Epididymitis

**DOI:** 10.3389/fsurg.2020.609661

**Published:** 2020-12-03

**Authors:** Mike Wenzel, Marina Deuker, Maria N. Welte, Benedikt Hoeh, Felix Preisser, Till Homrich, Volkhard A. J. Kempf, Michael Hogardt, Philipp Mandel, Pierre I. Karakiewicz, Felix K. H. Chun, Luis A. Kluth

**Affiliations:** ^1^Department of Urology, University Hospital, Goethe University Frankfurt am Main, Frankfurt, Germany; ^2^Cancer Prognostics and Health Outcomes Unit, Division of Urology, University of Montréal Health Center, Montréal, QC, Canada; ^3^German Society of Resisdents in Urology Academics Reconstructive Working Group, Planegg, Germany; ^4^Institute for Medical Microbiology and Infection Control, University Hospital Frankfurt, Frankfurt, Germany; ^5^University Center of Infectious Diseases, University Hospital Frankfurt, Frankfurt, Germany; ^6^University Center of Competence for Infection Control, Frankfurt, Germany

**Keywords:** epididymitis, suprapubic catheter, transurethral catheter, catheter, risk score, antibiotic treatment

## Abstract

**Objective:** This study aims to evaluate catheter management in acute epididymitis (AE) patients requiring inpatient treatment and risk factors predicting severity of disease.

**Material and Methods:** Patients with diagnosed AE and inpatient treatment between 2004 and 2019 at the University Hospital Frankfurt were analyzed. A risk score, rating severity of AE, including residual urine > 100 ml, fever > 38.0°C, C-reactive protein (CRP) > 5 mg/dl, and white blood count (WBC) > 10/nl was introduced.

**Results:** Of 334 patients, 107 (32%) received a catheter (transurethral (TC): *n* = 53, 16%, suprapubic (SPC): *n* = 54, 16%). Catheter patients were older, exhibited more comorbidities, and had higher CRP and WBC compared with the non-catheter group (NC). Median length of stay (LOS) was longer in the catheter group (7 vs. 6 days, *p* < 0.001), whereas necessity of abscess surgery and recurrent epididymitis did not differ. No differences in those parameters were recorded between TC and SPC. According to our established risk score, 147 (44%) patients exhibited 0–1 (*low-risk*) and 187 (56%) 2–4 risk factors (*high-risk*). In the high-risk group, patients received a catheter significantly more often than with low-risk (TC: 22 vs. 9%; SPC: 19 vs. 12%, both *p* ≤ 0.01). Catheter or high-risk patients exhibited positive urine cultures more frequently than NC or low-risk patients. LOS was comparable between high-risk patients with catheter and low-risk NC patients.

**Conclusion:** Patients with AE who received a catheter at admission were older, multimorbid, and exhibited more severe symptoms of disease compared with the NC patients. A protective effect of catheters might be attributable to patients with adverse risk constellations or high burden of comorbidities. The introduced risk score indicates a possibility for risk stratification.

## Introduction

Acute genitourinary infections, such as acute epididymitis, have a high prevalence in daily urological practice and can cause urosepsis and shock (1). The treatment consists of bed rest, scrotal elevation, analgesics, non-steroidal anti-inflammatory drugs, empirical antibiotics, as well as potential cause-specific interventions (1–3). Without appropriate treatment, acute epididymitis can lead to abscess formation and/or infertility (4). Therefore, it is important to identify appropriate initial management strategies to prevent complications and accelerate the recovery from acute epididymitis.

Acute epididymitis can be caused by migration of pathogens from the urethra or bladder. Thus, increased postvoid residual urine is a risk factor for acute epididymitis (1, 5). Urine drainage by catheter is the standard of care for patients with high residual urine or bladder outlet functional problems (6). Despite more historical reports that favored the effect of SPC over TC regarding urinary infection rates in patients with acute urinary retention, no studies exist that examined whether TC or SPC is superior in patients with acute epididymitis (7–9). Moreover, it is unknown whether applying a catheter accelerates the rate of recovery or improves the course of acute epididymitis.

In this retrospective study, we aimed to evaluate catheter management [catheter vs. no catheter (NC)] in patients presenting with acute epididymitis and inpatient treatment. Second, we aimed to assess outcomes between TC and SPC patients. Furthermore, we aimed to evaluate risk factors predicting severity and course of disease.

## Materials and Methods

### Patient Cohort

After approval of the ethic committee, all patients with an acute epididymitis and inpatient treatment between 2004 and 2019 were retrospectively identified in our institutional infectious data set. Inclusion criteria were male patients over 18 years who received inpatient hospital treatment of acute epididymitis. Exclusion criteria were known chronical epididymitis in patient's history or indwelling transurethral or suprapubic catheter at time of admission. Furthermore, all patients with an acute scrotum and an emergency operation due to a suspected testicular torsion were excluded in analyses.

### Clinical Evaluation and Catheter Management

Age, body mass index, comorbidities, and anticoagulant therapy were recorded to determine specific patient characteristics. In addition, inflammatory laboratory parameters of C-reactive protein (CRP) and white blood count (WBC) were recorded. After micturition, status of residual urine was defined by ultrasound-guided detection as residual urine of at least 50 ml. Temperature ≥38.0°C at time of admission was defined as fever. In inpatient setting, the time to nadir of CRP and WBC as well as the antibiotic therapy was evaluated. Furthermore, the length of inpatient stay (LOS), need of surgical intervention, and recurrence within 3 months was recorded. Additionally, microbiological analyses of the patients were recorded. For this purpose, urine dipstick at admission as well as urine and blood cultures with the corresponding pathogens and resistance spectrum were evaluated. Moreover, available information on prehospital antibiotic treatment in ambulatory settings, inhospital administration of antibiotics (oral vs. intravenous), and comedication with nonsteroidal anti-inflammatory drugs was also recorded.

Patients were divided in two main cohorts, catheter and NC. Further stratification according to TC and SPC was done. Indication for catheterization and inpatient treatment was based on clinical findings at presentation. Catheterization (TC or SPC) was based on physician's decision and patient's agreement.

### Risk Score for Severity of Disease

Based on the assessed clinical parameters, we established a clinical risk score reflecting the severity of disease consisting of four items that were routinely collected at admission (1 point for each matching parameter): ultrasound-guided residual urine, ≥100 ml; fever, ≥38.0°C; blood value of CRP, >5.0 mg/dl; and WBC, >10.0/nl. Already established, validated, and more differentiated infectious scores, e.g., SIRS criteria/SOFA score, could not be assessed due to missing variables at admission (breathing rates and carbon dioxide partial pressure in blood).

### Clinical Microbiology Analytics

All laboratory procedures were performed quality controlled (since the year 2010: laboratory accreditation according to ISO 15189:2007 standards; certificate number D-ML-13102-01-00, valid through 25 January 2021) using state-of-the-art technologies. Species identification was performed by matrix-assisted laser desorption ionization–time-of-flight analysis (VITEK MS; bioMérieux, Nürtingen, Germany). Antibiotic susceptibility testing was performed according to Clinical and Laboratory Standards Institute guidelines (and since 2019 via the more recent EUCAST guidelines) using VITEK 2 and/or antibiotic gradient tests (bioMérieux). Detection of genes encoding carbapenemases were routinely performed via polymerase chain reaction analysis and subsequent sequencing (e.g., including the bla genes for carbapenemases NDM, VIM, IMP, OXA-48 like, and KPC).

### Statistical Analysis

Descriptive statistics included frequencies and proportions for categorical variables. Means, medians, and interquartile ranges (IQR) were reported for continuously coded variables. The Chi-square tested for statistical significance in proportions' differences. The *t*-test and Kruskal-Wallis test examined the statistical significance of means' and medians' differences. In all statistical analyses, R software environment for statistical computing and graphics (R version 3.6.1) was used. All tests were two sided with a level of significance set at *p* < 0.05.

## Results

### Catheter vs. No Catheter

In total, 334 patients presenting and with acute epididymitis were included in our analysis. Median age was 48 years (IQR, 34–66). At admission for inpatient treatment, 107 (32%) and 227 (68%) of patients received a catheter vs. NC, respectively. Patient-specific characteristics at admission of patients with catheter and NC are displayed in Table 1. Overall, 82 (25%) patients presented with fever and 75 (23%) with residual urine. The median in hospital LOS was 6 days (IQR, 4–8).

**Table 1:** Patient characteristics of 334 patients with acute epididymitis and inpatient treatment at the University Hospital Frankfurt between 2004 and 2019 stratified by catheter management (catheter vs. no catheter) and risk score (low-risk vs. high-risk).

**Variable**	**Overall *n* = 334 (100%)**	**Catheter *n* = 107 (32%)**	**No catheter *n* = 227 (68%)**	***p*-value**	**0–1 Risk factors *N* = 147 (44.0%) *low-risk***	**2–4 Risk factors *N* = 187 (56.0%) *high-risk***	***p*-value**
Age; in years	48 (34–66)	59 (48–70)	42 (31–62)	<0.001	43 (31–62)	53 (40–69)	<0.001
Body Mass Index; in kg/m^2^	25.3 (23.6–27.6)	25.7 (24.2–28.8)	25.2 (23.2–26.7)	0.03	25.2 (23.8–27.2)	25.4 (23.5–27.8)	0.8
Diabetes mellitus	39 (12)	21 (20)	18 (8)	<0.01	13 (8.8)	26 (14)	0.2
Coronary heart disease	74 (22)	31 (29)	43 (19)	0.06	24 (16)	50 (27)	0.03
Benign prostate hyperplasia	45 (14)	25 (23)	20 (9)	<0.001	15 (10)	30 (16)	0.2
Anticoagulation therapy	66 (20)	31 (29)	35 (15)	<0.01	23 (16)	43 (23)	0.1
Previous recurrent infections	71 (21)	29 (27)	42 (19)	0.1	29 (20)	42 (23)	0.6
CRP >5 mg/dl at admission	168 (50)	63 (59)	105 (46)	0.04	17 (12)	151 (81)	<0.001
Time to CRP nadir; in days	2 (1–3)	2 (1–3)	2 (1–3)	1	2 (1–3)	2 (1–3)	0.04
WBC >10/nl at admission	212 (64)	78 (73)	134 (59)	0.02	40 (27)	172 (92)	<0.001
Time to WBC nadir; in days	2 (1–2)	2 (1–2)	1 (1–2)	0.4	2 (1–3)	1 (1–2)	<0.001
Fever at admission	82 (25)	28 (26)	54 (24)	0.7	3 (2)	79 (42)	<0.001
Residual urine at admission	75 (23)	39 (36)	36 (16)	<0.001	20 (14)	55 (29)	0.001
Suprapubic catheter at admission	54 (16)	54 (51)	0 (0)		13 (8.8)	41 (22)	<0.001
Transurethral catheter at admission	53 (16)	53 (49)	0 (0)		18 (12)	35 (19)	0.01
No catheter at admission	227 (68)	0 (0)	227 (100)		116 (79)	111 (59)	0.7
**Risk score**							
(one point for each parameter at admission):							
Residual urine ≥100 ml; fever ≥38.0°C; CRP >5.0 mg/dl; WBC>10.0/nl							
*Low–risk*: 0–1 points	147 (44)	31 (29)	116 (51)	<0.001	147 (100)	0 (0)	
*High–risk*: 2–4 points	187 (56)	76 (71)	111 (49)	0.01	0 (0)	187 (100)	
Length of stay; in days	6 (4–8)	7 (5–8)	6 (4–8)	<0.01	6 (4–8)	6 (5–8)	0.07
Abscess operation during treatment	54 (16)	22 (21)	32 (14)	0.2	21 (14)	33 (18)	0.5
Recurrent epididymitis within 3 months	19 (5.7)	8 (7.5)	11 (4.8)	0.5	9 (6.1)	10 (5.3)	0.9

*All values are median (interquartile range) or frequency (%)*.

*CRP, C reactive protein; WBC, White blood cells*.

*Risk Score (one point for each matching parameter): Residual urine ≥100ml, fever ≥38.0°C, blood value of CRP > 5.0 mg/dl and White Blood Cells (WBC) >10.0/nl*.

Stratification according to catheter vs. NC patients revealed significant differences: specifically, blood values of CRP [8.9 mg/dl (IQR, 3.5–15.9) vs. 4.4 mg/dl (IQR, 1.1–9.2)] and WBC [15/μl (IQR, 10.9–19.1) vs. 11.4/μl (IQR, 8.2–15.8)] (both *p* < 0.001) at admission were higher for catheter vs. NC group. Patients with catheter exhibited nitrite-positive dipstick and positive urine cultures significantly more frequently than NC group (nitrite, 38 vs. 21%, *p* = 0.02; urine culture, 61 vs. 33%, *p* < 0.001; Table 2). Furthermore, catheter patients harbored residual urine (36.4 vs. 15.9%, *p* < 0.001) and comorbidities [e.g., higher body mass index, diabetes, benign prostate hyperplasia (BPH), and anticoagulative therapy] significantly more often. The median LOS for inpatient treatment was shorter for patients with NC (6 days; IQR, 4–8), relative to the catheter group (7 days; IQR, 5–8; *p* < 0.01). No differences were recorded according to antibiotical treatment changes or escalation during inpatient stay, neither of abscess operation (21 vs. 14%, *p* = 0.2) or recurrences within 3 months (7.5 vs. 4.8%, *p* = 0.5).

**Table 2:** Microbiological characteristics of 334 patients with acute epididymitis and inpatient treatment at the University Hospital Frankfurt between 2004 and 2019 stratified by catheter management (catheter vs. no catheter) and risk score (*low-risk* vs. *high-risk)*.

**Variable**	**Overall *n* = 334 (100%)**	**Catheter *n* = 107 (32%)**	**No catheter *n* = 227 (68%)**	***p*-value**	**0–1 Risk factors *n* = 147 (44%) *low-risk***	**2–4 Risk factors *n* = 187 (56%) *high-risk***	***p*-value**
Nitrite in urine dipstick at admission	53 (28)	28 (38)	25 (21)	0.02	16 (21)	37 (32)	0.1
Urine culture taken	262 (78)	96 (90)	166 (73)	<0.001	103 (70)	159 (85)	<0.01
**RESULT OF URINE CULTURE**							
Negative urine culture	148 (57)	37 (39)	111 (67)	<0.001	76 (74)	72 (45)	<0.001
**BACTERIAL SPECIES OF POSITIVE URINE CULTURE**							
*Escherichia coli*	87 (76)	45 (76)	42 (76)	0.3	19 (70)	68 (78)	0.8
*Enterococcus faecalis*	4 (3.5)	1 (1.7)	3 (5.5)		1 (3.7)	3 (3.4)	
*Klebsiella spp*.	4 (1.5)	3 (5.1)	1 (1.8)		1 (3.7)	3 (3.4)	
*Pseudomonas spp*.	6 (2.3)	5 (5.2)	1 (0.6)		1 (3.7)	5 (5.7)	
Others	13 (11)	5 (8.5)	8 (15)		5 (19)	8 (9.2)	
**RESISTANCIES IN URINE CULTURE**							
Multidrug resistance	4 (1.5)	3 (3.1)	1 (0.6)	0.5	3 (2.9)	1 (0.6)	0.09
Fluoroquinolone resistance	2 (0.8)	2 (2.1)	0 (0)		0 (0)	2 (1.3)	
ß-Lactam resistance	9 (3.4)	5 (5.2)	4 (2.4)		2 (1.9)	7 (4.4)	
Other resistances	33 (13)	18 (19)	15 (9)		9 (8.7)	24 (15)	
Pathogens without any resistances	61 (23)	29 (30)	32 (19)		11 (11)	50 (31)	
Blood culture taken	73 (22)	38 (36)	35 (15)	<0.001	14 (9.5)	59 (32)	<0.001
**RESULTS OF BLOOD CULTURE**							
Negative blood culture	63 (86)	33 (87)	30 (86)	0.7	10 (71)	53 (90)	0.2
**BACTERIAL SPECIES OF POSITIVE BLOOD CULTURE**							
*Escherichia coli*	3 (30)	1 (20)	2 (40)	0.6	2 (50)	1 (17)	0.2
*Staphylococcus spp*.	3 (30)	1 (20)	2 (40)		1 (25)	2 (33)	
*Klebsiella* spp.	1 (20)	1 (20)	0 (0)		1 (25)	0 (0)	
Other	3 (30)	2 (40)	1 (20)		0 (0)	3 (50)	
Ambulatory antibiotic treatment prior in-hospital admission	80 (24)	20 (19)	60 (26)	0.2	40 (27)	40 (21)	0.3
**ANTIBIOTIC ADMINISTRATION**							
Oral	46 (14)	14 (13)	32 (14)	0.2	23 (16)	23 (12)	0.1
Intravenous	166 (50)	71 (66)	95 (42)		59 (40)	107 (57)	
Unknown	122 (36)	22 (21)	100 (34)		65 (44)	56 (31)	
**ANTIBIOTIC TREATMENT**							
Penicillins	77 (23)	43 (40)	34 (15)	<0.001	22 (15)	55 (29)	<0.01
Cephalosporins	104 (31)	28 (26)	76 (34)		54 (37)	50 (27)	
Carbapenems	5 (1.5)	2 (1.9)	3 (1.3)		4 (2.7)	1 (0.5)	
Fluoroquinolones	84 (25)	29 (27)	55 (24)		32 (22)	52 (28)	
Other	64 (19)	5 (4.7)	59 (26)		35 (24)	29 (16)	
**NSAID ADMINISTRATION**							
Yes	141 (42)	74 (69)	67 (30)	0.4	43 (29)	98 (52)	0.06
No	25 (7.5)	16 (15)	9 (4.0)		13 (8.8)	12 (6.4)	
Unknown	168 (51)	17 (16)	151 (66)		91 (62)	77 (42)	
Change of antibiotic regime within hospital treatment	97 (29)	37 (35)	60 (26)	0.2	39 (27)	58 (31)	0.4
Antibiotic escalation within hospital treatment	51 (15)	20 (19)	31 (14)	0.3	22 (15)	29 (16)	1

*All values are median (interquartile range) or frequency (%)*.

*NC, No catheter received; Spp, species; NSAID, Non-steroidal anti-inflammatory drugs*.

*Risk Score (one point for each matching parameter): Residual urine ≥100 ml, fever ≥38.0°C, blood value of CRP > 5.0 mg/dl and White Blood Cells (WBC) >10.0/nl*.

*Multidrug resistance: gram negative bacteria with resistancies against three of the four following antibiotic classes: Penicillins, Cephalosporins, Fluoroquinolones, Carbapenems*.

### Transurethral Catheter vs. Suprapubic Catheter

Of patients who received a catheter, 53 (49%) received a TC and 54 (51%) a SPC. No significant differences in age (*p* = 0.1), comorbidities (all *p* > 0.05), CRP (*p* = 0.4), WBC (*p* = 0.9), residual urine (*p* = 0.9), fever at admission (*p* = 0.4), LOS (*p* = 0.3), abscess operation during inpatient treatment (*p* = 0.3) nor recurrence of epididymitis (*p* = 0.3) were recorded. Likewise, the microbiological evaluation showed no significant differences regarding nitrite-positive dipsticks, positive urine and blood cultures, pathogens, resistances, and antibiotic escalation between TC and SPC (all *p* > 0.05).

### Clinical Risk Score

The distribution regarding the introduced risk score yielded 147 (44%) patients with 0–1 risk factors (*low-risk*) and 187 (56%) patients with 2–4 risk factors (*high-risk*) for severity of acute epididymitis (Table 1). Patients with a high-risk score were significantly older compared with low-risk patients (53 vs. 43 years, *p* < 0.001). Moreover, patients with a high-risk score received a catheter significantly more often than low-risk patients (TC, 22 vs. 9%, *p* < 0.001; SPC, 19 vs. 12%, *p* = 0.01). Furthermore, patients with low-risk score exhibited a negative urine culture significantly more often (74 vs. 45%, *p* < 0.001; Table 2). No differences within changes of antibiotic regime or escalation were recorded between low- and high-risk groups.

## Discussion

Until now, no recommendation exists according to catheter management in the treatment of acute epididymitis in the EAU guidelines or current epididymitis studies (1, 10, 11). The question whether patients with indications for inpatient treatment of acute epididymitis should receive a catheter or NC is still controversial and unacknowledged in daily urological practice.

In our cohort, patients with an acute epididymitis who received a catheter had a more severe disease at presentation, for example, with higher inflammation laboratory values, more often residual urine and comorbidities compared with patients with NC. There were no differences between the catheter vs. NC group according to complications (e.g., recurrences), required intervention and time to CRP and WBC nadir. However, our analyses showed some noteworthy findings:

Within daily urological practice, the indication for placing a catheter in men with acute epididymitis is the unobstructed release of potentially infectious urine. Furthermore, frequent argumentation for placing a SPC is the fully urine-free draining of the urethra. This is most likely explained by the lower urinary tract infection rates when comparing SPC vs. TC in BPH patients (12, 13). In comparison with patients that received NC, we found that patients who received a catheter at hospital admission exhibited a positive urine culture significantly more often. Conversely, compared with NC patients, patients with catheter did not exhibit longer time to CRP and WBC nadir, as well as more frequent antibiotic therapy escalation or pathogen resistances in urine culture. Moreover, patients with catheter only stayed 1 day longer in inpatient hospital treatment compared with NC patients. Thus, the significantly older and more comorbid patients who received a catheter did not show meaningful differences according to the recovery of the disease or LOS, as could have been expected due to age and comorbidity differences. In consequence, receiving a catheter (and draining the urethra) might accelerate the medical recovery in comorbid patients suffering severe epididymitis and shorten the time to discharge from inpatient to ambulant treatment. Additionally, according to BPH and residual urine, these patients were significantly more prevalent in the catheter group. Studies by May et al. and Truzzi et al. demonstrated association between residual urine caused by functional bladder outlet problems as BPH and urinary tract infections (14, 15). This leads to the assumption that patients with acute epididymitis, BPH, and residual urine are more likely to harbor severe infections. In consequence, our data can be interpreted that for patients, who harbored nitrite-positive urinary tract infections at admission, receiving a catheter is a necessary part in a multimodal therapy concept. This applies especially to older patients with comorbidities, such as bladder outlet problems, in order to accelerate recovery rates and discharging from inpatient treatment (5).

Second, according to comparison of SPC vs. TC in acute epididymitis patients, our analyses recorded no differences in patient characteristics, inpatient treatment, and microbiological analysis, nor in recurrence rates. To the best of our knowledge, serval studies exist that compared catheters (TC vs. SPC) in different urological diseases/procedures, but there is no evidence claiming which type of catheter patients should receive in the management of inpatient treatment of acute epididymitis (16–18). It is assumed that in acute prostatitis, SPC may prevent disease chronification in comparison with TC (19). Yoon et al. suspected that a TC applies higher pressure on the prostatic ducts in the urethra, and this could explain the higher rates of chronification in acute prostatitis patients with TC relative to SPC (19). This thesis cannot be confirmed in the case of acute epididymitis since the ductus deferentes, which are in contact with the epididymis, lead into the prostatic urethra. This could be interpreted as an indication that the form (SPC or TC) of the catheter in acute epididymitis is less important than in acute prostatitis, as long as a catheter is used. Nonetheless, it has to be mentioned that the exact benefit of a catheter and the different types in the treatment of acute epididymitis can only be assessed to a limited extent retrospectively. Since our evaluation did not reveal any significant differences between TC and SPC, it may be helpful to identify different risk profiles that may benefit more from catheter insertion. For this reason, the applied risk score was introduced in order to make more precise differentiations between NC vs. catheter as well as TC vs. SPC patients.

Third, according to the applied risk score, we found that patients with a high-risk score exhibited significantly different characteristics than those with a low-risk score. Screening and risk assessment is mandatory for the initial evaluation of patients with acute infections or (pre-)urosepsis conditions in the emergency department to plan the appropriate treatment and scoring systems, such as the SOFA score or SIRS criteria that are well-established at intensive care units or in academic infectious analyses (1, 20, 21). Nonetheless, in daily urological emergency practice, these scores are often collected incompletely, due to the lack of documentation of the necessary breathing rates or carbon dioxide partial pressure in the blood, which is not part of routine urological assessment. Hongo et al. found that WBC and CRP level, as well as fever are predictors for severe epididymitis in in- and outpatient setting (11). Despite this study, no urological risk score exists in the literature for inpatient acute epididymitis. We addressed this void and used four easily available parameters to create a suitable urological score for risk stratification of patients with acute epididymitis, containing the predictors of Hongo et al.

Furthermore, Truzzi et al. already published a threshold of 180 ml residual urine for exhibiting a positive urine culture with a sensitivity of 87% and specify of 98.5%. Conversely, May et al. could not confirm this threshold but also indicated a relation between residual urine and urinary tract infections (14, 15). However, these findings made it important to include residual urine within our risk score. In our analysis, patients in the high-risk group had positive urine cultures more frequently. This can be explained by the fact that patients with a high-risk score exhibited residual urine significantly more often. Moreover, patients with a high-risk score (2–4 points) were significantly older than low-risk patients (0–1 points) and suffered from cardiac diseases more often. Additionally, patients with a high-risk score of acute epididymitis received a TC or SPC more frequently than low-risk patients. Interestingly, urine drainage by catheter led to the same median LOS for patients with high- and low-risk score of an acute epididymitis (high-risk 6 days vs. low-risk 6 days). Additionally, the fact of no significant differences according to abscess operations during inpatient treatment or recurrences of epididymitis after 3 months between high-risk and low-risk patients could be explained by the fact that high-risk patients receive catheters significantly more frequent.

Our study has several limitations. First, it is based on retrospective data. Second, it is not known how many patients refused a catheter in the emergency management of acute epididymitis, nor is the physician bias known, which could have led to a bias in the compared cohorts. Third, it was not known how many acute epididymitis were related to sexually transmitted diseases as no specific microbiological analyses were performed to investigate sexually transmitted disease-related epididymitis. Fourth, admission of patients with acute epididymitis was not standardized and based on objective criteria. The introduced risk score as well as the comparison of NC vs. catheter groups should be evaluated in prospective trials.

Taken together, it can be concluded that using a catheter in older and multimorbid patients with acute epididymitis or with a high amount of risk factors nearly equalizes the length of inpatient treatment compared with younger and fitter patients who did not receive a catheter. Therefore, catheters should be considered a part of multimodal treatment of acute epididymitis in daily practice when patients with severe acute epididymitis are seen in the emergency department. Moreover, our introduced risk score can easily be applied to assess severity of acute epididymitis and may provide clinicians with a helpful tool to distinguish patients who are more likely to benefit from catheter usage in emergency settings of acute epididymitis.

## Data Availability Statement

The raw data supporting the conclusions of this article will be made available by the authors, without undue reservation.

## Ethics Statement

The studies involving human participants were reviewed and approved by Ethic committee University Hospital Frankfurt, number 19-234. Written informed consent for participation was not required for this study in accordance with the national legislation and the institutional requirements.

## Author Contributions

MW and MD: manuscript writing/editing, protocol/project development, and data analysis. MNW: manuscript writing/editing. BH and FP: data analysis. TH: data collection or management. VK, MH, and PM: manuscript writing/editing. PK: data analysis and manuscript writing/editing. FC: protocol/project development. LK: protocol/project development and manuscript writing/editing. All authors contributed to the article and approved the submitted version.

## Conflict of Interest

The authors declare that the research was conducted in the absence of any commercial or financial relationships that could be construed as a potential conflict of interest.
